# Butyric Acid-Modified m-P14 Peptide Ameliorates Anti-Glomerular Basement Membrane Disease

**DOI:** 10.3390/ijms27114810

**Published:** 2026-05-27

**Authors:** Nan Jiang, Yan-Lun Gu, Huang Kuang, Zhao Cui, Ming-Hui Zhao, Xiao-Cong Pang, Xiao-Yu Jia

**Affiliations:** 1 Renal Division, Peking University First Hospital, No. 8 Xishiku Street, Xicheng District, Beijing 100034, China; 2311110262@stu.pku.edu.cn (N.J.);; 2 Institute of Nephrology, Peking University, Beijing 100034, China; 3 Key Laboratory of Renal Disease, Ministry of Health of China, Beijing 100034, China; 4 Key Laboratory of Chronic Kidney Disease Prevention and Treatment, Ministry of Education of China, Beijing 100034, China; 5 Department of Pharmacy, Peking University First Hospital, No. 8 Xishiku Street, Xicheng District, Beijing 100034, China; 6 State Key Laboratory of Natural and Biomimetic Drugs, School of Pharmaceutical Sciences, Peking University, Xue Yuan Rd. 38, Haidian District, Beijing 100191, China

**Keywords:** anti-glomerular basement membrane disease, glomerulonephritis, peptides, pharmacokinetics, immunotherapy

## Abstract

The non-collagenous domain 1 of the α3 chain of type IV collagen (α3(IV)NC1) is the primary autoantigen in anti-glomerular basement membrane (anti-GBM) disease. We previously developed a modified antigen-specific peptide, m-P14, derived from the nephritogenic epitope α3_127–148_, which ameliorated experimental anti-GBM nephritis. However, its short half-life limits clinical translation. This study evaluated a butyrate-conjugated derivative (m-P14-BA) to improve pharmacokinetic properties while preserving therapeutic efficacy. M-P14-BA and m-P14 were administered to α3_127–148_ immunized Wistar Kyoto rats in early and late treatment settings. Renal injury parameters and intrarenal inflammation were assessed, and pharmacokinetic profiles were evaluated following intraperitoneal administration in beagle dogs. M-P14-BA reduced proteinuria, crescent formation, glomerular IgG deposition, complement activation, and inflammatory cell infiltration, with overall efficacy comparable to m-P14 in early treatment settings. In late treatment settings, m-P14-BA was associated with a significant improvement in blood urea nitrogen levels and modest reductions in proteinuria and histopathological injury. Butyrate conjugation markedly improved pharmacokinetics, prolonging plasma elimination half-life by approximately 2.8-fold and increasing systemic exposure nearly fourfold. These pharmacokinetic improvements were associated with maintained therapeutic efficacy at a reduced dose, with 10 mg/kg m-P14-BA achieving effects broadly similar to those observed with 30 mg/kg m-P14. In summary, butyrate conjugation improves the pharmacokinetic profile of an antigen-specific therapeutic peptide while preserving therapeutic activity, suggesting a potential strategy to enhance the translational feasibility of peptide-based immunotherapy in anti-GBM disease.

## 1. Introduction

Anti-glomerular basement membrane (GBM) disease is a rare and severe autoimmune kidney disorder characterized by the presence of circulating anti-GBM antibodies and linear deposition of these antibodies along the GBM [1]. Clinically, anti-GBM disease typically presents as rapidly progressive glomerulonephritis, frequently accompanied by pulmonary hemorrhage [2,3]. Kidney pathology shows diffuse crescent formation in glomeruli, with linear IgG deposition along the glomerular capillary walls on immunofluorescence [1]. Conventional treatment for anti-GBM disease includes a combination of plasma exchange and immunosuppressive agents to remove pathogenic autoantibodies [4,5]. Although current treatments have significantly improved patient survival, the one-year kidney survival rate remains poor, ranging from 15% to 60% [5]. Exploring alternative or adjunctive therapies is crucial for improving kidney outcomes in anti-GBM disease.

The primary target antigen of anti-glomerular basement membrane (anti-GBM) disease resides within the non-collagenous domain 1 (NC1) of the α3 chain of type IV collagen in the GBM [6,7]. Although autoantibodies have been shown to be pathogenic, increasing evidence indicates that T cell-mediated immune injury, particularly by T helper 17 (Th17) cells, plays a critical role in the pathogenesis of this disease. Antigen-specific CD4^+^ T cells alone can induce renal injury [8] and crescentic glomerulonephritis [9,10]. In our previous studies, a linear epitope peptide, α3_127–148_, was identified on α3(IV)NC1, which encompasses the EB conformational epitope [6] and is recognized by autoreactive T and B cells from patients with anti-GBM disease [11,12]. Subsequently, a modified peptide, m-P14, was designed by replacing a critical pathogenic residue [13]. Treatment with m-P14 significantly ameliorated kidney injuries in a rat model of anti-GBM disease by decreasing Th17 cells and enhancing the ratio of Tregs/Th17 cells, showing promising therapeutic effects as an antigen-specific immunomodulatory reagent. Although m-P14 exhibits immunomodulatory effects, its poor solubility and short half-life necessitate further chemical modifications.

Short-chain fatty acids (SCFAs), primarily acetate, propionate, and butyrate, play a key role in regulating immune responses by modulating the differentiation, recruitment, and activation of immune cells [14,15,16]. In experimental autoimmune encephalomyelitis (EAE), a model of multiple sclerosis (MS), SCFAs induce a tolerogenic and anti-inflammatory T cell phenotype [17]. A clinical study in MS demonstrated that oral administration of propionate significantly reduced relapse rates and ameliorated brain lesions, accompanied by an increase in regulatory T cells (Tregs) and a marked decrease in Th17 cells [18]. Our previous study also showed that oral supplementation with short-chain fatty acids (SCFAs), especially butyrate, alleviated kidney injuries by regulating T cell differentiation [19]. Therefore, we designed a modified m-P14 peptide by conjugating it with butyrate and hypothesized that this modification could improve the solubility and extend the half-life of m-P14, thereby reducing the therapeutic dose and enhancing efficacy while maintaining its biological activity. In this study, we evaluated the pharmacokinetic characteristics and therapeutic efficacy of a butyrate-conjugated m-P14 peptide (m-P14-BA) in a rat model of anti-GBM disease.

## 2. Results

### 2.1. m-P14-BA Treatment Effectively Alleviates Kidney Injuries in Anti-GBM Nephritis

**Figure 1:**
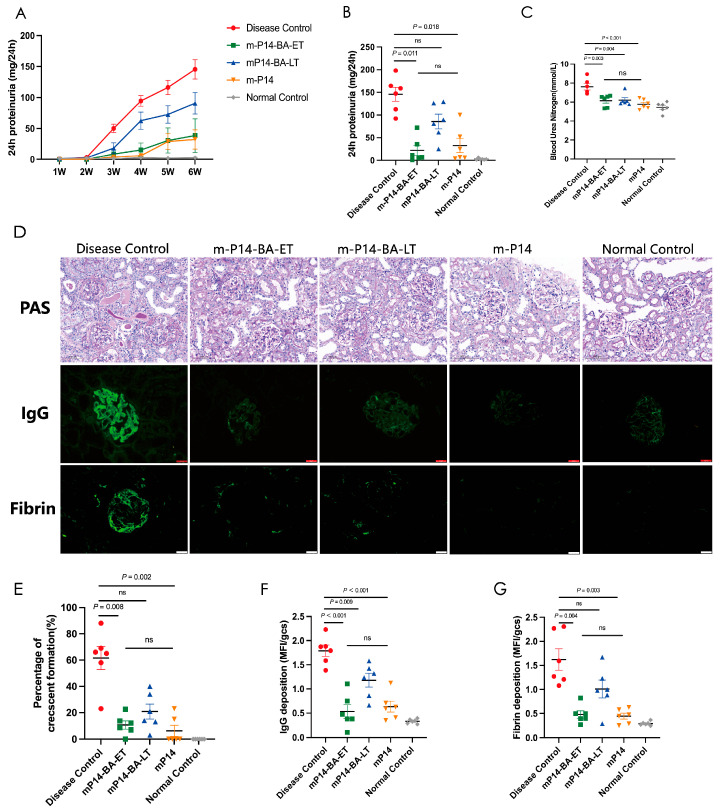
Clinical and kidney pathological features in experimental anti-GBM nephritis treated with m-P14-BA and m-P14. Treatment with m-P14 (30 mg/kg) or low-dose m-P14-BA (10 mg/kg) was initiated on the day of immunization. Urinary protein levels progressively increased over time in the disease control group, whereas they were significantly reduced in the early treatment groups (**A**). At week 6, early treatment with m-P14 (30 mg/kg) or low-dose m-P14-BA (10 mg/kg) achieved comparable reductions in urinary protein and blood urea nitrogen (BUN), and late treatment with low-dose m-P14-BA (10 mg/kg) also significantly lowered BUN levels with a trend toward decreased urinary protein levels (**B**,**C**). Compared with the disease control group, kidney pathology was markedly ameliorated in the treatment groups (**D**). Early treatment with m-P14 (30 mg/kg) as well as m-P14-BA (10 mg/kg) significantly reduced crescent formation (**E**), glomerular IgG deposition (**F**), and fibrin deposition (**G**). The effects of early treatment with m-P14 and m-P14-BA on renal pathological outcomes were broadly comparable. Glomerular IgG and fibrin deposition were quantified as mean fluorescence intensity (MFI) per glomerular cross-section. For each animal, five randomly selected glomeruli were analyzed, and the mean value was used for statistical analysis. MFI, mean fluorescence intensity; gcs, glomerular cross-section. Data are presented as mean ± SEM; *n* = 6 rats per group.

Proteinuria appeared in the disease group around weeks 3–4 following immunization with α3_127–148_ peptide and increased as the disease progressed (Figure 1A). In comparison with the disease control group, early treatment with m-P14 at a dose of 30 mg/kg significantly reduced urinary protein excretion and blood urea nitrogen (BUN) levels at week 6 (urinary protein: 32.38 ± 15.48 vs. 145.60 ± 15.51 mg/24 h, *p* = 0.018, Figure 1B; BUN: 5.78 ± 0.21 vs. 7.61 ± 0.33 mmol/L, *p* < 0.001, Figure 1C). Early treatment with m-P14-BA at a lower dose of 10 mg/kg also resulted in a significant reduction in both urinary protein and BUN compared with the disease control group (urinary protein: 22.0 ± 11.2 vs. 145.60± 15.51 mg/24 h, *p* = 0.011; BUN: 6.15± 0.25 vs. 7.61 ± 0.33 mmol/L, *p* = 0.003). The efficacy of m-P14-BA in lowering urinary protein and BUN was comparable to that of m-P14, with no statistically significant difference between the two groups. In addition, later treatment with m-P14-BA at 10 mg/kg significantly reduced blood urea nitrogen (BUN) levels at week 6 (6.22 ± 0.25 vs. 7.61 ± 0.33 mmol/L, *p* = 0.004) compared with the disease control group, while urinary protein levels showed a trend toward reduction.

Compared with the disease control group, early treatment with m-P14 (30 mg/kg) and m-P14-BA (10 mg/kg) significantly reduced crescent formation (m-P14: 6.3 ± 4.1% vs. 61.5 ± 8.7%, *p* = 0.002; m-P14-BA: 10.7 ± 3.1% vs. 61.5 ± 8.7%, *p* = 0.008; Figure 1E). Both glomerular IgG deposition and fibrin deposition were markedly decreased (IgG: m-P14, 0.6 ± 0.1 vs. disease control, 1.8 ± 0.1, *p* < 0.001; m-P14-BA, 0.5 ± 0.1 vs. disease control, 1.8 ± 0.1, *p* < 0.001; Figure 1F; fibrin: m-P14, 0.4 ± 0.1 vs. disease control, 1.6 ± 0.2, *p* = 0.003; m-P14-BA, 0.5 ± 0.1 vs. disease control, 1.6 ± 0.2, *p* = 0.004; Figure 1G). No significant differences were observed between m-P14 and m-P14-BA treatment groups in crescent formation, IgG deposition, or fibrin deposition. In addition, compared with the disease control group, subsequent treatment with m-P14-BA (10 mg/kg) also significantly reduced glomerular IgG deposition (IgG: m-P14-BA, 1.2 ± 0.1 vs. 1.8 ± 0.1 in the disease control group, *p* = 0.009). Crescent formation and fibrin deposition also showed decreasing trends. Taken together, these findings suggest that early intervention with a lower dose of m-P14-BA provides renoprotective effects broadly comparable to those of m-P14 in experimental anti-GBM glomerulonephritis.

### 2.2. m-P14-BA Treatment Inhibits Intramolecular Epitope Spreading Induced by α3_127–148_

**Figure 2:**
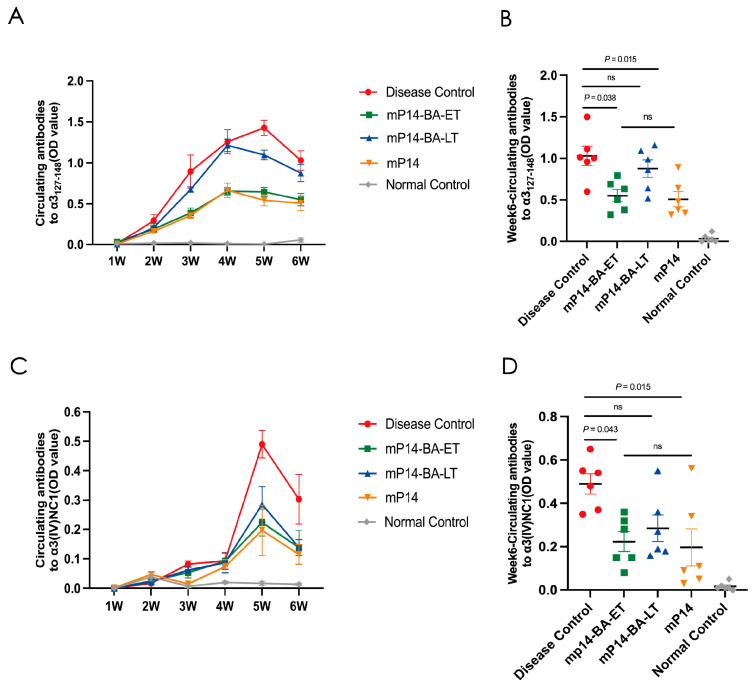
Comparison of circulating anti-α3_127–148_ and anti-α3(IV)NC1 antibody levels among rats immunized with α3_127–148_. From week 1 to week 6 after immunization (**A**) and at week 6 (**B**), circulating anti-α3_127–148_ antibody levels were significantly reduced in rats treated with m-P14 (30 mg/kg) or low-dose m-P14-BA (10 mg/kg). Similarly, circulating anti-α3(IV)NC1 antibody levels were also significantly decreased in both treatment groups (**C**,**D**). Data are presented as mean ± SEM; *n* = 6 rats per group.

ELISA was performed to evaluate circulating antibody levels and epitope specificity after treatment with m-P14-BA or m-P14. By week 6, both the disease control and treatment groups had developed autoantibodies targeting the immunogen α3_127–148_ peptide (Figure 2A). Early treatment with m-P14 (30 mg/kg) or low-dose m-P14-BA (10 mg/kg) resulted in comparable antibody levels, which were significantly lower than those in the disease control group (m-P14: 0.5 ± 0.1 vs. disease control:1.0 ± 0.1, *p* = 0.015; m-P14-BA: 0.6 ± 0.1 vs. disease control:1.0 ± 0.1, *p* = 0.038; Figure 2B). In the disease control group, all rats exhibited intramolecular epitope spreading by the presence of circulating antibodies against the full-length α3(IV)NC1 protein (Figure 2C,D). At week 6, early intervention with either m-P14 (30 mg/kg) or low-dose m-P14-BA (10 mg/kg) also significantly reduced anti-α3(IV)NC1 antibody levels compared with the disease control group (m-P14: 0.2 ± 0.09 vs. disease control: 0.5 ± 0.05, *p* = 0.015; m-P14-BA: 0.2 ± 0.05 vs. disease control: 0.5 ± 0.05, *p* = 0.043; Figure 2D).

### 2.3. m-P14-BA Inhibits the Intra-Kidney Inflammatory Responses

**Figure 3:**
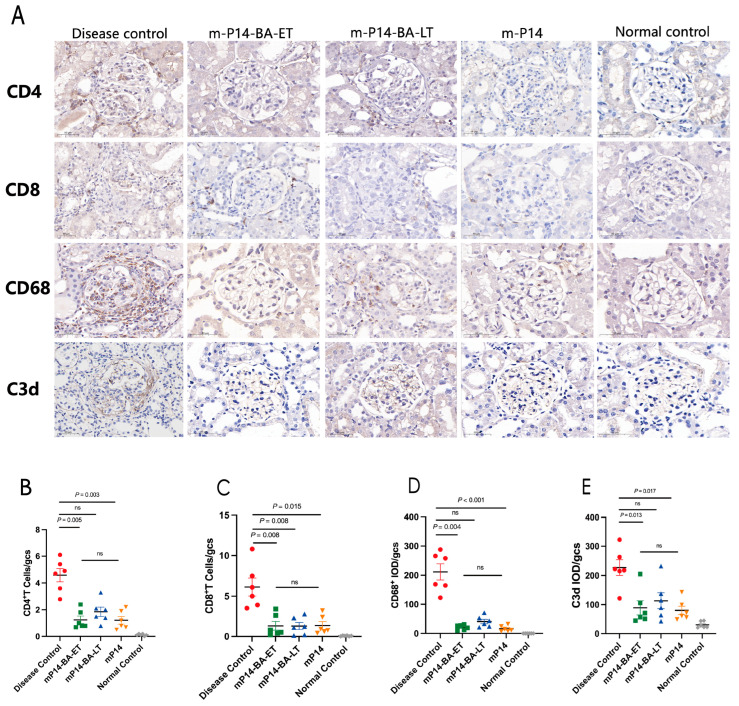
m-P14-BA suppressed the intra-kidney inflammatory responses. Infiltration of CD4^+^ T cells, CD8^+^ T cells, CD68^+^ macrophages, and complement C3d deposition were observed in the kidneys of rats with anti-GBM glomerulonephritis (**A**). CD4^+^ and CD8^+^ T-cell infiltration was quantified as the number of positively stained cells per glomerular cross-section (cells/gcs), whereas CD68^+^ macrophage infiltration and C3d deposition were quantified as integrated optical density per glomerular cross-section (IOD/gcs). At least 10 consecutive glomeruli were analyzed for each marker at ×400 magnification using Image-Pro Plus software. Consistent thresholding and analysis criteria were applied across all groups to minimize measurement bias. Data are presented as mean ± SEM; *n* = 6 rats per group. Early treatment with m-P14 (30 mg/kg) and early treatment with low-dose m-P14-BA (10 mg/kg) significantly reduced CD4^+^ T-cell infiltration (**B**), CD8^+^ T-cell infiltration (**C**), CD68^+^ macrophage infiltration (**D**), and C3d deposition (**E**). No significant differences were observed between m-P14 and m-P14-BA in reducing renal inflammatory cell infiltration and complement deposition. IOD, integrated optical density; gcs, glomerular cross-section. Data are presented as mean ± SEM; *n* = 6 rats per group.

The intra-kidney infiltration of CD4^+^ and CD8^+^ T cells and macrophages was significantly reduced in m-P14 or m-P14-BA intervention groups, compared with the α3_127–148_ immunized group (Figure 3A–E). In the disease control group, there was marked intra-kidney infiltration of complement, T cells, and macrophages (Figure 3A). Compared with the disease controls, early treatment with m-P14 (30 mg/kg) or low-dose m-P14-BA (10 mg/kg) significantly reduced intra-kidney infiltration of CD4^+^ T cells (m-P14: 1.2 ± 0.3 vs. disease control: 4.6 ± 0.5, *p* = 0.003; m-P14-BA: 1.3 ± 0.3 vs. disease control: 4.6 ± 0.5, *p* = 0.005; Figure 3B), CD8^+^ T cells (m-P14: 1.4 ± 0.5 vs. disease control: 6.1 ± 1.1, *p* = 0.015; m-P14-BA: 1.3 ± 0.5 vs. disease control: 6.1 ± 1.1, *p* = 0.008; Figure 3C), and macrophages (m-P14: 16.2 ± 4.0 vs. disease control: 211.5 ± 27.6, *p* < 0.001; m-P14-BA: 20.8 ± 3.5 vs. disease control: 211.5 ± 27.6, *p* = 0.004; Figure 3D), as well as glomerular complement C3d deposition (m-P14: 80.0 ± 14.5 vs. disease control: 227.0 ± 26.8, *p* = 0.017; m-P14-BA: 89.2 ± 24.8 vs. disease control: 227.0 ± 26.8, *p* = 0.013; Figure 3E). No significant differences were observed between the m-P14 and m-P14-BA treatment groups. Similarly, compared with the disease control group, later treatment with a low dose of m-P14-BA (10 mg/kg) significantly reduced renal infiltration of CD8^+^ T cells (1.3 ± 0.3 vs. 6.1 ± 0.5, *p* = 0.008; Figure 3B). Reductions in CD4^+^ T-cell infiltration (Figure 3C) and macrophage infiltration (Figure 3D) were also observed, although these changes did not reach statistical significance. In addition, glomerular C3d deposition showed a trend toward reduction (Figure 3E).

### 2.4. Pharmacokinetic Characterization of m-P14-BA and m-P14

**Figure 4:**
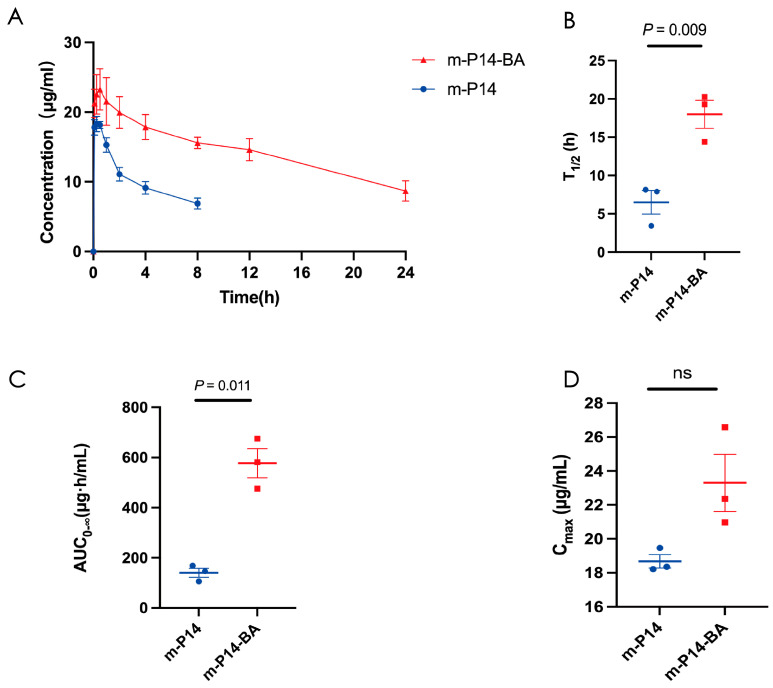
Pharmacokinetic evaluation of the peptides in Beagle dogs. Pharmacokinetic profiles of m-P14-BA and m-P14 in beagle dogs. (**A**) Plasma concentration–time curves of m-P14-BA and m-P14 following a single intraperitoneal administration at a dose of 10 mg/kg, with plasma peptide concentrations measured by LC–MS/MS at the indicated time points over 24 h. (**B**) Plasma elimination half-life (T_1_/_2_) of m-P14-BA and m-P14. (**C**) Systemic exposure of m-P14-BA and m-P14 expressed as the area under the plasma concentration–time curve from time zero to infinity (AUC_0_–∞). (**D**) Peak plasma concentration (Cmax) of m-P14-BA and m-P14.

The pharmacokinetic profiles of m-P14-BA and m-P14 were evaluated in beagle dogs following a single intraperitoneal administration at a dose of 10 mg/kg, with serial blood sampling over 24 h. Plasma concentrations of both peptides were quantified by LC–MS/MS and are shown in Figure 4A. Pharmacokinetic analysis demonstrated that butyric acid modification significantly prolonged the in vivo circulation of m-P14. As shown in Figure 4B, the plasma elimination half-life (T_1_/_2_) of m-P14-BA was markedly increased compared with that of the parent peptide m-P14 (18.0 h vs. 6.5 h, *p* = 0.009). Consistent with the prolonged half-life, systemic exposure to m-P14-BA was substantially enhanced, as reflected by a nearly four-fold increase in AUC_0_–∞ compared with m-P14 (577.3 ± 99.6 vs. 140.2 ± 31.9 μg·h/mL, *p* = 0.011; Figure 4C). In contrast, the peak plasma concentration (Cmax) did not differ significantly between m-P14-BA and m-P14 (Figure 4D), indicating that butyric acid conjugation primarily affected peptide elimination rather than initial systemic absorption.

## 3. Discussion

Our findings demonstrate that conjugation of the short-chain fatty acid butyrate to the antigen-specific therapeutic peptide m-P14 prolongs its in vivo half-life and is associated with maintained therapeutic efficacy at a reduced dose. To the best of our knowledge, this is among the first studies to explore short-chain fatty acid modification as a strategy to optimize peptide therapy in autoimmune kidney disease, providing a potential approach for improving peptide drug design.

Peptide-based immunotherapy has emerged as a promising treatment strategy for autoimmune diseases owing to its ability to regulate antigen-specific immune responses and restore immune homeostasis [20,21,22]. To date, multiple self-antigen-derived peptides have entered clinical trials for diseases such as multiple sclerosis and celiac disease [23,24,25]. For example, a modified glutamic acid decarboxylase 65 (GAD65)-derived peptide formulated with alum adjuvant (GAD-alum) delayed disease progression and preserved C-peptide secretion in patients with recent-onset type 1 diabetes [26]. Similarly, in multiple sclerosis, a mixture of myelin basic protein-derived peptides (ATX-MS-1467) reduced gadolinium-enhancing brain lesions and promoted IL-10-secreting regulatory T cells in experimental models [27,28]. These studies collectively support the concept that self-antigen-derived peptides can reestablish antigen-specific immune tolerance while minimizing the adverse effects associated with nonspecific immunosuppression [13]. In our present study, we investigated a modified self-antigen peptide targeting α3(IV)NC1 that attenuates autoimmune kidney injury by modulating T cell differentiation, thereby extending the application of peptide-based immunotherapy to anti-GBM disease. Our findings demonstrate that antigen-specific immune modulation represents a feasible therapeutic strategy with potential translational relevance.

Importantly, butyric acid conjugation did not appear to alter the previously established immunomodulatory mechanism of m-P14 in anti-GBM disease [13]. Consistent with our prior work, administration of m-P14-BA resulted in reduced proteinuria, improved renal histopathology, and attenuated intrarenal inflammatory responses. At the immunological level, m-P14-BA treatment effectively reduced antibody responses against both the immunizing epitope α3_127–148_ and the intact α3(IV)NC1 protein, indicating suppression of epitope spreading. In anti-GBM disease, crescentic glomerulonephritis (CGN) is the defining pathological feature reflecting severe glomerular injury. In the present study, the marked reduction in crescent formation observed in the early treatment groups was consistent with the overall improvements in renal injury and immune responses described above. These concordant findings suggest that the reduction in crescent formation reflects a global attenuation of disease activity rather than an isolated pathological change. Although N-terminal modification may theoretically influence B-cell epitope recognition and antibody binding, the use of a flexible KKKKK-Ahx linker was intended to provide spatial separation and minimize steric interference. Consistent with this design, the observed reduction in antigen-specific antibody responses suggests that the core antigenic structure was largely preserved, although this was not directly evaluated in the present study. Further functional characterization of immune responses in the future, including cytokine profiling and T cell phenotyping, would help to better define the underlying mechanisms. These findings suggest that butyrate conjugation preserves the antigen-specific therapeutic mechanism of m-P14 while enabling further optimization of its pharmacological properties.

An important finding of the present study is that butyric acid conjugation markedly improved the pharmacokinetic behavior of m-P14 without compromising its therapeutic efficacy. Compared with the parent peptide, m-P14-BA exhibited a substantially prolonged plasma elimination half-life and significantly increased systemic exposure, as reflected by an approximately 3-fold increase in T_1/2_ and a nearly 4-fold elevation in AUC_0_–∞. Notably, these pharmacokinetic advantages were associated with therapeutic activity at a reduced dose, with 10 mg/kg m-P14-BA achieving effects broadly similar to those observed with 30 mg/kg m-P14 in the anti-GBM rat model. The dissociation between systemic exposure and peak plasma concentration further suggests that butyric acid conjugation primarily affects peptide elimination rather than initial absorption, thereby enabling more sustained antigen-specific immune modulation over time. In the present study, plasma samples were collected up to 48 h post-administration, because drug concentrations at 48 h fell below the lower limit of quantification of our LC–MS method. This may limit precise characterization of the terminal elimination phase. It should also be noted that pharmacokinetic data obtained in beagle dogs are primarily intended to reflect relative changes in systemic exposure and half-life, and direct extrapolation to dosing strategies in rats is therefore limited.

The relative contributions of pharmacokinetic and pharmacodynamic factors to the observed therapeutic effects warrant further consideration. Based on the current data, the improved therapeutic performance of m-P14-BA is most consistently explained by enhanced pharmacokinetic properties, including prolonged circulation time and increased systemic exposure, which facilitate sustained antigen-specific immune modulation. Importantly, we did not observe evidence suggesting a fundamental alteration in the antigen-specific mechanism of action compared with the parent peptide. Thus, the therapeutic effects observed at the reduced dose are more likely attributable to pharmacokinetic enhancement rather than a definitive increase in intrinsic biological activity or target engagement. Nevertheless, a contributory role of pharmacodynamic effects, either directly or through local release of butyrate, cannot be excluded and warrants further investigation. In addition, the lack of a dose-matched comparison with the parent peptide represents a limitation of the present study. Compared with emerging broad-spectrum therapies such as IgG-degrading enzymes (e.g., imlifidase), which rapidly reduce circulating IgG levels within hours through enzymatic cleavage [29], antigen-specific approaches may offer the advantage of selectively targeting pathogenic immune responses while preserving overall immune function, although the onset of action may be relatively gradual. Taken together, the observed efficacy at a reduced dose of m-P14-BA is more likely to reflect improved systemic stability and prolonged exposure rather than an intrinsic enhancement of the biological activity of the peptide itself.

Chemical modification of therapeutic peptides represents a well-established strategy to overcome pharmacokinetic limitations. Common approaches include lipidation [30], cyclization [31], the incorporation of D-amino acids [32] and N-methylation [33], all of which aim to enhance stability and prolong in vivo exposure. Although lipidation with medium- or long-chain fatty acids has been successfully applied to peptide drugs such as semaglutide, the use of short-chain fatty acids for peptide modification has not been previously explored in this context [34]. Butyrate, a short-chain fatty acid, is known to influence T cell differentiation [35,36] and exert immunoregulatory effects in autoimmune diseases including multiple sclerosis [37] and asthma [38]. In our previous work, oral administration of free butyrate significantly ameliorated experimental anti-GBM nephritis; however, the dose of butyrate required in those studies was approximately 150-fold higher than the amount covalently conjugated to m-P14 in the present study, indicating that the therapeutic effects observed here are primarily attributable to the modified peptide rather than free butyrate. Nevertheless, the independent contribution of the butyrate moiety remains to be fully defined. Short-chain fatty acids can regulate T-cell homeostasis through mechanisms such as GPCR signaling and histone deacetylase (HDAC) inhibition. Therefore, although the markedly lower butyrate dose in the conjugated form supports a predominantly peptide-driven mechanism, a contributory role of locally or systemically released butyrate cannot be excluded. Further studies are required to delineate the relative contributions of the conjugated peptide and free butyrate to the overall therapeutic effects.

The limited efficacy observed in the later treatment setting likely reflects the rapid establishment of structural glomerular injury in anti-GBM disease. Once extensive antibody deposition and crescent formation occur, renal damage becomes less reversible and less responsive to antigen-specific immunomodulation. Nevertheless, the significant reduction in blood urea nitrogen and the trends toward decreased proteinuria and crescent formation indicate that m-P14-BA retains partial therapeutic activity after disease establishment. These findings highlight the importance of early intervention and suggest that pharmacokinetic optimization may help extend the therapeutic window of antigen-specific peptide therapy.

Although the precise mechanisms underlying the improved pharmacokinetic profile of m-P14-BA remain to be fully elucidated, enhanced metabolic stability and altered interactions with plasma components following butyric acid conjugation may contribute to its reduced clearance. In addition, short-chain fatty acids possess favorable aqueous solubility compared with medium- and long-chain fatty acids, which may further facilitate peptide handling and bioavailability.

From a translational perspective, the improved pharmacokinetic profile of m-P14-BA may enhance the clinical feasibility of antigen-specific peptide therapy by prolonging systemic exposure and reducing dosing frequency. However, several limitations should be acknowledged. First, the pharmacokinetic analysis was performed in beagle dogs, whereas therapeutic efficacy was evaluated in a rat model, and additional species-specific studies are needed before clinical translation. Second, the intraperitoneal route used in the present study may not directly reflect clinically preferred routes of administration. Moreover, long-term safety, immunogenicity, and optimal dosing frequency remain to be further evaluated. Therefore, the current findings should be regarded as proof-of-concept evidence supporting further preclinical development rather than direct evidence for immediate clinical application.

In conclusion, our study suggests that short-chain fatty acid conjugation may improve the pharmacokinetic properties and translational feasibility of an antigen-specific immunomodulatory peptide for autoimmune kidney disease. These findings support m-P14-BA as a candidate for further preclinical development and highlight SCFA-based peptide modification as a potential strategy for optimizing tolerance-inducing peptides.

## 4. Materials and Methods

### 4.1. Animals and Immunization

**Figure 5:**
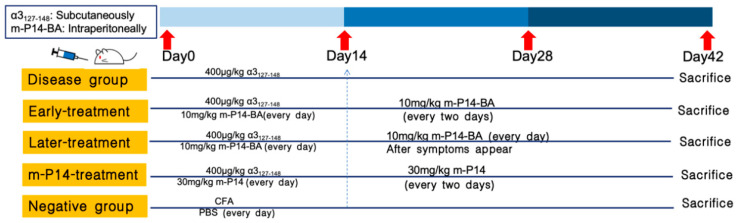
Modified m-P14 peptide interventions in experimental anti-GBM glomerulonephritis. All WKY rats were immunized with 400 μg/kg α3_127–148_ on day 0 via subcutaneous injection into the hind footpad, *n* = 6 rats per group. Early treatment groups received m-P14 30 mg/kg or m-P14-BA 10 mg/kg via daily i.p. injection from day 0–14, then every other day from day 15–28. Rats were observed for two additional weeks from day 29–42; the key time points (days 0, 14, 28, and 42) are indicated with red arrows. Late treatment of m-P14-BA 10 mg/kg was initiated upon detection of hematuria or proteinuria. Negative controls received CFA on day 0. All rats were sacrificed on day 42.

As previously described [13,39], female WKY rats (4–5 weeks old; Vital River Laboratories, Beijing, China) were immunized by footpad injection with α3_127–148_ peptide (0.4 mg/kg) emulsified in complete Freund’s adjuvant (CFA; Sigma-Aldrich, St. Louis, MO, USA). Control animals received CFA alone. Twenty-four-hour urine samples were collected weekly using metabolic cages, and blood samples were obtained by angular venipuncture. Rats were sacrificed at week 6 after immunization (*n* = 6 per group), and kidney and blood samples were collected for analysis. A schematic diagram of the experimental design is shown in Figure 5.

The pharmacokinetic profiles of m-P14-BA and m-P14 were evaluated in male beagle dogs (*n* = 3, 6–8 months old). For the single-dose PK study, three dogs received an intraperitoneal injection of m-P14 at a dose of 10 mg/kg. Dose conversion between rats and dogs was performed using body surface area-based allometric scaling as previously described [40]. Following a washout period of two weeks, the same animals received an intraperitoneal injection of m-P14-BA at an equivalent dose. Serial blood samples (200 μL) were collected at 5, 15, and 30 min, and at 1, 2, 4, 8, 12, and 24 h post-administration. Blood samples were collected into heparinized tubes and centrifuged at 12,000 rpm for 15 min to obtain plasma.

The experiment was approved by the Experimental Animal Ethics Committee of Peking University First Hospital (Beijing, China), approval number: 2023109.

### 4.2. Synthesis of Peptides

α3_127–148_, m-P14 and Butyric acid-modified m-P14 (m-P14-BA) (Table 1) were synthesized on an automatic peptide synthesizer using F-moc chemistry (Beijing Scilight Biotechnology Ltd., Co., Beijing, China), and purified by reverse-phase CIS column on a preparative HPLC. A KKKKK-Ahx linker was introduced to connect the butyrate moiety to the parent peptide. The poly-lysine segment (KKKKK) was used to improve solubility, while Ahx (6-aminohexanoic acid) served as a flexible spacer to provide spatial separation and reduce potential steric hindrance [41,42]. This design was intended to optimize physicochemical properties without altering the antigenic structure of the peptide. Purified peptides were analyzed by HPLC for purity and mass spectrometry for correct sequence. Peptides with purity over 98%were used for further tests.

### 4.3. Treatment Designs

Anti-GBM nephritis was induced in Wistar Kyoto (WKY) rats by active immunization with the pathogenic T cell epitope α3_127–148_ [13]. Rats were randomly assigned to groups using computer-generated random numbers (Figure 1), *n* = 6 rats per group. For early-treatment group, m-P14 (30 mg/kg) or m-P14-BA (10 mg/kg) was administered intraperitoneally daily from day 0 to 14, and then every other day from day 15 to 28. PBS was used as vehicle control in the disease control group. For later treatment group, mP14-BA was administered intraperitoneally daily at 10 mg/kg upon the onset of disease, approximately 2 weeks after immunization. Pharmacokinetic analysis was conducted.

### 4.4. Evaluation of Kidney Injuries

Urinary protein and blood urea nitrogen levels were measured using an automatic biochemical analyzer (Unicel DxC 600 Synchron, Beckman Coulter, Brea, CA, USA). For histological analysis, kidneys were fixed in 10% neutral buffered formalin, processed, paraffin-embedded, sectioned at 3 μm, and stained with periodic acid–Schiff (PAS). At least 50 glomeruli per sample were examined by light microscopy to assess renal lesions.

For immunofluorescence staining, frozen kidney sections (5 μm) were fixed in acetone and incubated with FITC-conjugated anti-rat IgG (Jackson ImmunoResearch Laboratories Inc., Seattle, WA, USA) at 1:50. Fibrin deposition was detected on acetone-fixed frozen kidney sections using FITC-conjugated rabbit anti-rat fibrin antibody (DAKO, Santa Clara, CA, USA). For immunofluorescence quantification, glomerular IgG and fibrin deposition were analyzed using Image-Pro Plus software (version 6.0; Media Cybernetics, Dallas, TX, USA). IgG and fibrin signals were quantified as mean fluorescence intensity (MFI) per glomerular cross-section. For each animal, five randomly selected glomeruli were analyzed for each marker, and the mean value was used for statistical analysis.

### 4.5. Detection of Serum Antibodies by Enzyme-Linked Immunosorbent Assays (ELISA)

Serum antibodies against peptides were detected by ELISA [39]. Plates were coated overnight with α3127–148 peptide (6 μg/mL) or α3 (2 μg/mL). Serum samples diluted 1:100 in PBST were incubated at 37 °C for 1 h. After washing, alkaline phosphatase-conjugated goat anti-rat IgG (1:5000; Sigma) was added for 30 min. The reaction was developed using p-nitrophenyl phosphate substrate, and absorbance was measured at 405 nm.

### 4.6. Intra-Kidney Infiltration and Deposits by Immunohistochemistry

Immunohistochemistry was performed to detect CD4^+^ T cells, CD8^+^ T cells, CD68^+^ macrophages and C3d deposition. Formalin-fixed, paraffin-embedded kidney sections (4 μm) underwent heat-induced antigen retrieval (CD4, CD8, CD68, C3d) or enzymatic retrieval for CD68 (0.04% pepsin). Endogenous peroxidase was blocked with 3% H_2_O_2_, followed by blocking with 3% BSA. Sections were incubated overnight at 4 °C with primary antibodies against CD4 (1:400, Cell Signaling Technology, Danvers, MA, USA), CD8 (1:100, Santa Cruz Biotechnology, Dallas, TX, USA), CD68 (1:100, Abcam, Cambridge, MA, USA) or C3d (1:20, R&D Systems, Minneapolis, MN, USA), followed by horseradish peroxidase-conjugated secondary antibodies (ZGSB-BIO, Beijing, China). Signals were visualized using 3,3′-diaminobenzidine.

For quantification, at least 10 consecutive glomeruli were analyzed for each marker at ×400 magnification using Image-Pro Plus software. CD4^+^ and CD8^+^ T-cell infiltration was quantified as the number of positively stained cells per glomerular cross-section (cells/gcs), as individual positive cells could be clearly identified. Because CD68^+^ macrophages were more abundant and often densely distributed within glomeruli, CD68^+^ macrophage infiltration was quantified as integrated optical density per glomerular cross-section (IOD/gcs), which better reflects the overall CD68^+^ staining burden. C3d deposition was also quantified as integrated optical density per glomerular cross-section (IOD/gcs), as it represents a deposition signal rather than discrete cells. The same threshold and analysis parameters were applied consistently across all groups.

### 4.7. Pharmacokinetic Analysis

For pharmacokinetic analysis, plasma samples (50 μL) were mixed with internal standard (α3_127–148_ peptide, 5 μg/mL) and acetonitrile for protein precipitation. After centrifugation at 12,000 rpm for 20 min at 4 °C, the supernatant was collected and analyzed by liquid chromatography–tandem mass spectrometry (LC–MS/MS).

LC–MS/MS analysis was performed using a SCIEX Triple Quad™ 7500 (AB Sciex, Framingham, MA, USA) mass spectrometer coupled to a SCIEX ExionLC™ AD system (AB Sciex, Framingham, MA, USA). Separation was achieved on a Waters BEH C18 column (2.5 μm, 2.1 mm × 100 mm) using a gradient of 0.1% formic acid in water (A) and acetonitrile (B). Mass spectrometric parameters are shown in Table 2.

### 4.8. Statistical Analyses

Data are presented as mean ± SEM. Statistical analyses were performed using GraphPad Prism 10.0 (GraphPad Software, Boston, MA, USA). Differences among groups were analyzed using one-way ANOVA followed by Dunnett’s multiple comparisons test or the Kruskal–Wallis test followed by Dunn’s multiple comparisons test, as appropriate. Treatment groups were compared with the disease control group. A two-tailed *p* value < 0.05 was considered statistically significant.

## 5. Conclusions

This study suggests that butyrate conjugation improves the pharmacokinetic properties of the antigen-specific therapeutic peptide m-P14 and supports therapeutic activity at a reduced dose in experimental anti-GBM disease. These findings indicate that short-chain fatty acid conjugation may represent a potential strategy to improve the translational feasibility of peptide-based antigen-specific immunotherapy. Further studies are needed to evaluate the safety, immunogenicity, dosing route, and translational potential of m-P14-BA.

## Figures and Tables

**Table 1:** Peptide names and sequences.

Peptide Name	Sequence
α3_127–148_ (P14)	TDIPPCPHGWISLWGFSFIMF
m-P14	TDIPPCPHGWSSLWKGFSFIMF
m-P14-BA	Butyric acid-KKKKK-Ahx-TDIPPCPHGWSSLWKGFSFIMF

**Table 2:** Parameters for the detection of m-P14 and m-P14-BA using LC–MS/MS with multiple reaction monitoring.

	m-P14-BA	m-P14	α3_127–148_ (IS)
Spray voltage	1500 V	1500 V	1500 V
Source temperature	300 °C	300 °C	300 °C
MRM transition (*m*/*z*)	847.00 → 772.52	853.70 → 1115.00	861.00 → 1126.00
Dwell time	100 ms	100 ms	100 ms
Entrance Potential	10.0 V	10.0 V	10.0 V
Collision Energy	34.0 V	27.0 V	32.0 V
Collision Cell Exit Potential	18.0 V	20.0 V	20.0 V

## Data Availability

All data are available from the corresponding authors upon reasonable request.
